# Involvement of Aquaporins in the Pathogenesis, Diagnosis and Treatment of Sjögren’s Syndrome

**DOI:** 10.3390/ijms19113392

**Published:** 2018-10-30

**Authors:** Muhammad Shahnawaz Soyfoo, Clara Chivasso, Jason Perret, Christine Delporte

**Affiliations:** 1 Department of Rheumatology and Physical Medicine, Hôpital Erasme, Université Libre de Bruxelles, Brussels 1070, Belgium; msoyfoo@ulb.ac.be; 2 Laboratory of Pathophysiological and Nutritional Biochemistry, Université Libre de Bruxelles, Brussels 1070, Belgium; clara.chivasso@ulb.ac.be (C.C.); jason.perret@ulb.ac.be (J.P.)

**Keywords:** aquaporins, Sjögren’s syndrome, therapy, salivary glands, lacrimal glands

## Abstract

Sjögren’s syndrome (SS) is a chronic autoimmune disease characterized by lymphocytic infiltration of salivary and lacrimal glands resulting in diminished production of saliva and tears. The pathophysiology of SS has not yet been fully deciphered. Classically it has been postulated that sicca symptoms in SS patients are a double step process whereby lymphocytic infiltration of lacrimal and salivary glands (SG) is followed by epithelial cell destruction resulting in keratoconjunctivitis sicca and xerostomia. Recent advances in the field of the pathophysiology of SS have brought in new players, such as aquaporins (AQPs) and anti AQPs autoantibodies that could explain underlying mechanistic processes and unveil new pathophysiological pathways offering a deeper understanding of the disease. In this review, we delineate the link between the AQP and SS, focusing on salivary glands, and discuss the role of AQPs in the treatment of SS-induced xerostomia.

## 1. Introduction

Sjögren’s syndrome (SS) is a chronic systemic autoimmune disease characterized by lympho-plasmocytic infiltration of exocrine glands and, more specifically, salivary and lacrimal gland [1]. As such, SS is classified as primary when occurring alone and secondary when associated with another autoimmune disease such as rheumatoid arthritis or systemic lupus erythematous. The resulting features of the marked infiltration and destruction of salivary and lacrimal glands define SS as sicca syndrome with xerostomia and keratoconjunctivitis. Beyond the classic sicca symptoms, systemic involvement of other organs can occur [1]. The protean features of SS make the diagnosis of SS relatively difficult. Many SS patients remain undiagnosed or have the diagnosis established many years after the onset of the symptoms. However, early diagnosis and classification of this disease is particularly important for enabling appropriate diagnostic evaluation and optimization of therapeutic intervention [2]. The diagnosis and the classification of patients are not based on the presence of single pathognomonic signs or symptoms. The disease is usually identified by the presence of a combination of clinical and laboratory manifestations. These manifestations include the classification criteria of the disease, which usually also serve as diagnostic criteria. Ideally, the classification criteria should only be used as diagnostic criteria when their sensitivity and specificity are both close to 100%. In practice, this is rather difficult in many instances, particularly at the disease onset, when the disease manifestations are not fully overt, and when the diagnosis depends largely on the ability and the expertise of the physician. However, the use of universally-accepted classification criteria for SS has created a consensus between clinical researchers, facilitating the standardization of the diagnosis in patients taking part in multicenter clinical studies, thereby enabling the analysis of results in a common and unbiased fashion. Recently, a new set of classification criteria for SS has been proposed [3]. It is worth mentioning that there is currently no diagnostic marker of SS, even though some prognostic markers have been proposed.

Despite of the staggering progress made to unearth the different inherent processes underscoring the pathophysiology of sicca symptoms in SS, the initial triggering event remains unknown [4,5]. Different mechanisms have been delineated in portraying the salivary dysfunction in SS, whereby epithelial cells play a central pivotal role [6]. In SS, there is an impairment of the function, as well as a destruction, of the salivary gland (SG) epithelial cells. Epithelial cells, per se, have protean features and functions beyond the sheer and simple defining characteristic of organizing and maintaining the architecture of the SG. Indeed, they also act as non-professional antigen-presenting cells participating actively in the immune processes underlying the pathogenesis of SS. As such, there is an increased expression of CD40 and adhesion molecules such as intercellular adhesion molecule 1 (*ICAM 1*), as well as *B7* co -stimulation molecules, thereby enhancing the activation of CD4+ T cells, HLA type-II molecules. Moreover, following the activation of the epithelial cells, there is an increased local production of chemokines and pro inflammatory cytokines and B-cell activating factors. The aftermath of the activation of the epithelial cells and ensuing production of immunoactive molecules foster lymphoid cells homing and antigen cell presentation, thereby, in a vicious circle, amplify the interactions between epithelial cells and immune cells [7]. Furthermore, following interferon type 1 secretion, there is also secretion of B-cell activating factor by the activated epithelial cells, thereby promoting B-cells activation and proliferation [5,8]. These well-defined sequences of immune activation, leading to aberrant lymphocyte homing, unrestrained pro inflammatory cytokine production, and the corollary of SG disorganization and destruction, clearly delineate and promote the predominant role of the epithelial cells as key to the development of SS, hence the term *auto-immune epithelitis* [9,10].

The mechanisms responsible for salivary gland hypofunction and the corollary of xerostomia are not fully deciphered, but there is sufficient compelling evidence to substantiate the role of salivary gland destruction due to the autoimmune underpinnings in SS as described above. Moreover, there are also several lines of proofs undergirding the fact that dry mouth and dry eyes do not solely result from gland destruction, and that other mechanisms, including the presence of anti-muscarinic autoantibodies, altered mucin expression, nitric oxide-mediated salivary gland dysfunction, and modified aquaporin-5 (AQP5) distribution are also potential active players responsible for the sicca syndrome. In this review, we describe the involvement of aquaporins (AQPs) in the pathogenic features of SS, focusing on salivary glands, and the potential diagnostic and therapeutic possibilities of AQPs in SS.

## 2. Expression and Function of AQPs in Salivary Glands

**Figure 1:**
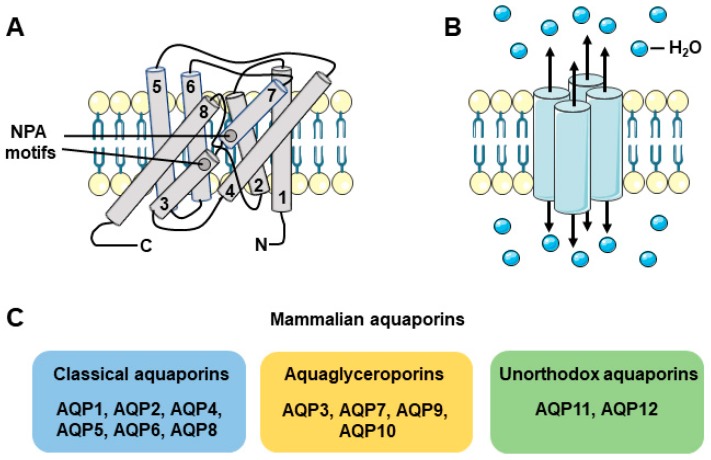
Characteristics and classification of AQPs. **A**: AQPs are made of six transmembrane helices and two short helices containing the NPA motifs for the classical AQPs and aquaglyceroporins, or NPC motifs for unorthodox AQPs. **B**: AQPs need to associate as tetramers to be functional. **C**: AQPs are subdivided into the classical AQPs, the aquaglyceroporins and the unorthodox AQPs.

AQPs, a family of water-permeable channels, are small transmembrane proteins of about 28 kDa, implicated in transcellular water permeability in all living organisms [11]. AQPs are made of six transmembrane helices and two short helices comprising each a canonical Asparagine-Proline-Alanine (NPA) motif (Figure 1A). The AQP monomers need to associate as tetramers to be functional [12] (Figure 1B). It is now well established that transcellular water fluxes occur through both diffusion and a facilitated pathway mediated by AQPs [12]. Water diffusion occurs at relatively low velocity and volume, while transcellular water movement through AQPs occurs at much higher volumes to cross membranes at a much higher velocity. In most tissues, AQPs-mediated water flow is directed by osmotic gradients and osmosis. So far, 13 mammalian AQPs have been identified [13,14]. These AQPs are classified into three subfamilies according to their permeability features and sequences homologies. The subfamilies include: (a) the classical AQPs only permeable to water (AQP0, AQP1, AQP2, AQP4, AQP5, AQP6, and AQP8); (b) the aquaglyceroporins permeable to water as well as to small uncharged molecules, such as glycerol and urea (AQP3, AQP7, AQP9, and AQP10), and (c) unorthodox AQPs, whose permeability still remains to be clearly established (AQP11 and AQP12) [12,13,14] (Figure 1C).

**Figure 2:**
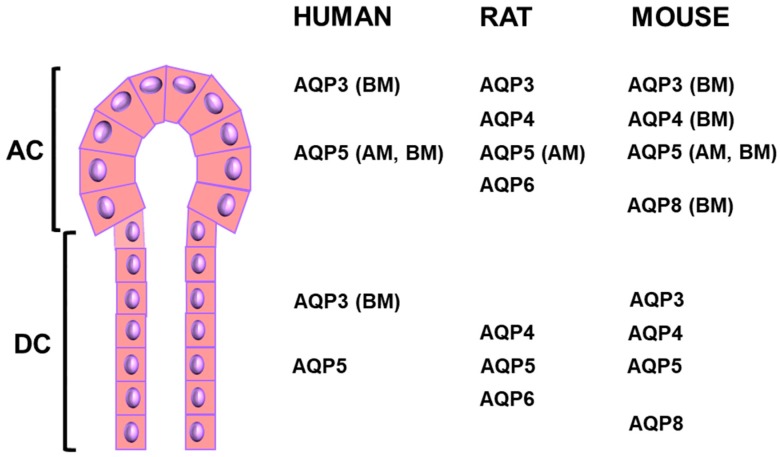
Localization of AQPs in salivary gland acinar and ductal cells. AC: acinar cells; AM: apical membrane; BM: basolateral membrane; DC: ductal cells.

Of the known AQPs, six are expressed in mammalian SG [15]. AQP1 is expressed in human and mouse endothelial cells as well as human and mouse myoepithelial cells, but not in acinar and ductal salivary gland cells [16]. AQP3 is detected in human, rat, and mouse salivary gland acinar cells, and is mainly located at their basolateral membranes in human and mouse [16,17]. AQP3 is also expressed in human and mouse salivary gland ductal cells, with a basolateral localization in humans [16]. AQP4 is expressed in rat and mouse salivary gland acinar and ductal cells, and localized at the basal membrane in mouse acinar cells [16]. In addition, in human SG, AQP4 is expressed at the basolateral membrane of acinar cells, apical and lateral membranes of ductal cells, and in myoepithelial cells [18]. AQP5 has been detected in rat and mouse parotid, submandibular and sublingual glands, as well as in human parotid, submandibular, sublingual and minor labial SG [15,16,19]. AQP5 is predominantly found at the apical membranes of the human, rat, and mouse salivary gland acini, but also at the basolateral membrane in humans and mice [16,17,19]. The localization of AQP5 to ductal cells is somehow controversial. Indeed, most of the studies analyzing AQP5 protein expression in the SG did not detect the presence of AQP5 in rat submandibular ductal cells, while a few studies have reported the presence of AQP5 in the apical membranes of intercalated ducts in submandibular glands and in the intercellular ducts of parotid. The detectable ductal expression of AQP5 protein is in contradiction with the water-impermeable characteristics of SG ductal cells [20]; this is basically accounted for the current salivary secretion model [21,22]. Further studies are required to fully appreciate the role of AQP5 in SG ductal cells. A naturally-occurring point mutation in the rat *AQP5* gene influenced its level of expression in acinar cells and the pilocarpine-induced salivary secretion [23] without affecting its intrinsic water permeability [24]. However, this *AQP5* mutation has not been observed in humans. AQP6 is localized to secretory granule membranes of rat salivary gland acinar cells and in rat ductal cells [16]. AQP8 is expressed in rat salivary gland myoepithelial cells, as well as mouse salivary gland acinar (basolateral membrane) and ductal cells. Despite the presence of several AQPs in SG, only AQP5 has been validated to functionally contribute to salivary secretion using knockout mice. Indeed, pilocarpine-induced salivary secretion was reduced by about 60% in AQP5 knockout mice as compared to wild-type mice, and saliva was more viscous and hypertonic [25,26]. In addition, transcellular water permeability was reduced by 65% and 77% in parotid and sublingual acinar cells subjected to osmotic challenges [26]. Pilocarpine-induced salivary flow was not impaired in AQP1, AQP4, and AQP8 knockout mice, ruling out a role of these AQPs in the basal and regulated salivary secretion mechanisms [25,27,28]. The putative involvement of AQP3 in saliva secretion remains to be assessed. In the current salivary secretion model, AQP5 contributes to transcellular water flow to the acini lumen in response to formation of a transepithelial osmotic gradient created by NaCl accumulation within the acini lumen [21,22]. Figure 2 summarizes the expression of AQPs in SG.

## 3. Defective Localization of AQPs in the Salivary Glands of SS

Defective localization of AQP5 has been extensively demonstrated in salivary glands of SS patients and SS mice models [16,29]. In both human and mice SG, AQP5 distribution was significantly altered from a predominant apical fashion in controls to a strikingly substantial basolateral membrane distribution in SS [17,30,31,32,33,34]. In SG from some SS patients, AQP5 distribution was also shown to not be altered [35,36,37]. However, in SG from mice models displaying SS, it was shown that the modification of expression of AQP5 protein could be related to the degree of inflammatory cells within the SG [38,39]. As such, the modification of AQP5 distribution depicted in some SG of SS patients could be related to the degree of inflammation, an intrinsic feature of the pathophysiological mechanism inherent to SS. It could therefore be speculated that the functional interplay of different pro-inflammatory cytokines, which have been advocated to contribute to the inflammatory infiltrates in the SG of SS patients, be at least responsible for the modification of AQP5 localization [40]. The principal pro-inflammatory cytokines identified in SS patients are the interferons α and γ (IFNα, IFN γ), interleukin 17 (IL-17), interleukin 7 (IL-7), Tumor necrosis factor α (TNF-α), interleukin 1β (IL-1β), and B-cell activating factor (BAFF) [41,42,43]. Type-I interferons (IFNs) play a cardinal role in the pathogenesis of SS [44,45]. Upon activation, cytosolic DNA sensors interact with the stimulator of interferon genes (STING) protein, and the ensuing activation of STING causes increased expression of *type-I IFN-α* [46]. IL-17 also has a pivotal involvement in SS where overexpression in mice models triggers an enhanced inflammatory response accompanied by salivary impairment [47]. On the other side of the coin, IL-17 downregulation resulted in significantly dampened inflammatory responses and improved salivary function in SS mice models [48]. IL-7 plays a key role in T cell development and homeostasis, and is associated with SG inflammation and salivary hypofunction in SS mice models [49]. TNFα, a pro-inflammatory cytokine produced by several cell types including CD4+ T cells and epithelial cells, promotes SS pathogenesis by promoting immune cells recruitment [50]. IL1ß also plays a central role in the pathogenesis of SS [51]. B-cell activating factor (BAFF) is involved in the B-cells response, i.e., antibody secretion, following antigen presentation. BAFF promotes B-cell proliferation, maturation and survival, and is primarily induced by type-I and type-II interferons [52]. Animal models for SS have been of particular help in deciphering the role played by proinflammatory cytokines in the pathogenesis of the disease [53,54,55].

Interestingly, several compelling lines of evidence support the hypothesis that modification of AQP5 localization is linked to inflammation. Neutralization of IFN-γ markedly improved salivary secretion and *AQP5* expression in anti-programmed death ligand 1-treated non-obese diabetic (NOD)/ShiLtJ mice [56]. In addition, while IFNα upregulated AQP5 expression in human parotid gland cells ex vivo [57], STING activation led to SS in mice [58]. Whether STING activation leads to altered AQP5 localization in SG stills remains to be addressed. While IL-7 has been shown to be involved in the pathogenesis of SS, further studies are required to assess whether IL-7 alters AQP5 localization in SG. Blockage of IL-7 receptors led to increased levels of AQP5 and improved the manifestations of SS in mice [59]. Indeed, the injection of anti-TNF antibodies to NOD mice reduced SG inflammatory foci and increased AQP5 protein expression [50]. Inactivation of TGFß receptor I resulted in increased proinflammatory cytokines expression and altered AQP5 localization in mice SG [60]. Rituximab was shown to increase apical AQP5 localization in SG acinar cells in one SS patient [61]. In addition, activation of the G-protein coupled formyl peptide receptor 2 (ALX/FPR2) by the lipid mediators lipoxin A_4_ and resolvin D1 (RvD1) plays a protective role against inflammation in mice SG as ALX/FPR2 knockout mice displayed enhanced inflammatory responses to lipopolysaccharides as well as a significant decrease in M3 muscarinic receptors and AQP5 protein expression and saliva secretion [62].

Under normal conditions, AQP5 translocates to the apical membrane after activation of muscarinic and adrenergic receptors. From a physiological point of view, it could be speculated that abnormal localization of AQP5 results from a defective activation of muscarinic M3 receptor or a modified protein-protein interaction ensuing aberrant translocation of AQP5. Whether the abnormal distribution of AQP5 is linked to the presence of autoantibodies to M3 receptor is an open question that remains to be addressed. It is also interesting to highlight the fact that a naturally-occurring point mutation of AQP5 has been detected in rats, but not in humans, and induces reduced salivary secretion and decreased AQP5 protein expression [23].

In SG from SS patients, AQP3 protein expression was increased at the apical membrane of acinar cells [17], while AQP1 [63] and AQP4 [18] protein expression were decreased in myoepithelial cells. In a patient suffering from SS, Rituximab increased AQP1 protein expression in myoepithelial cells and salivary flow [61]. Taking into account these data, and the fact that acetylcholine induces myoepithelial cells contraction and AQP1 trafficking, it was inferred that AQP1 might be involved in saliva secretion [61]. However, the hypothesis was not supported by data obtained with AQP1 knockout mice [27,28]. Further studies are necessary to better appreciate the role of AQPs in salivary hypofunction occurring in SS patients.

## 4. Autoantibodies against AQP in SS

Antibodies against AQP4 and AQP1 have been consistently observed in the sera from patients with neuromyelitis optica (NMO) [64,65,66]. Neuromyelitis optica, also known as Devic’s syndrome, is a rare autoimmune disease affecting the central nervous system. NMO spectrum disorders (NMOSD) are sometimes found in association with other autoimmune disorders, including SS [67]. The prominent fact that antibodies against AQP4 have been found in the majority of patients suffering from NMOSD differentiates the latter from other demyelinating diseases such as multiple sclerosis [68]. The role of AQP4 has been shown to be cardinal underlying the physiopathogenesis of NMOSD [69,70]. AQP4 is the most widely expressed AQP on the astrocytic foot processes at the blood brain barrier, as well as on the subependymal and subapial regions. There is substantial extensive loss of AQP4 protein expression in the astrocytes of patients suffering from NMOSD. Recent studies have also shown that antibodies against AQP4 play an active pathogenic role in the destruction and dysfunction of astrocyte [69]. More recently, several antibodies against AQP1, AQP3, AQP5, AQP8, and AQP9 have been detected in patients suffering from SS. AQP1 autoantibodies were detected in 27.7% of SS patients and none in controls [71]. Autoantibodies against AQP5 were also observed in a cohort of SS patients, and were related to significantly lower basal salivary secretion rates [72]. The role of these autoantibodies against AQP5 has been recently deciphered. Indeed, these autoantibodies bind to the extracellular loops of AQP5 and hamper water flux. This observation in itself could explain the role of AQP5 autoantibodies in the pathogenesis underlying the sicca syndrome. Furthermore, other autoantibodies against AQP1, AQP3, AQP8, and AQP9 have been observed in patients with SS [71]. Autoantibodies against AQP8 and AQP9 were detected with a higher frequency (39%) whilst autoantibodies against AQP1 and AQP3 were observed at a significantly lower frequency [71]. Interestingly, no autoantibodies were observed in healthy patients. Further studies should investigate the impact of autoantibodies against AQPs, and in particular against AQP5, on SG integrity and function. In NMOSD, AQP4-IG blocking peptides are currently under investigation as potential therapy. The principle underlying this proof of concept is that the non-pathogenic anti-AQP4 antibody (aquaporumab) is in direct competition with pathogenic AQP4-IgG for binding to AQP4 on astrocytes [73]. This proof of concept could represent the first forays for treating patients with SS and autoantibodies against aquaporins.

## 5. Role of AQPs in the Treatment of Sjögren’s Syndrome

**Figure 3:**
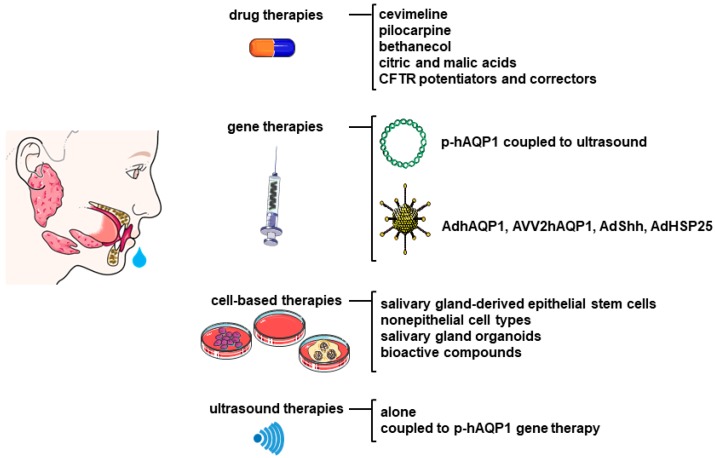
Role of AQPs in the treatment of xerostomia in SS patients. AAV2hAQP1: adeno-associated virus type 2 encoding hAQP1; AdhAQP1: adenovirus encoding hAQP1; AdShh: adenovirus encoding sonic hedgehog; AdHSP25: adenovirus encoding heat shock protein 25; CFTR: cystic fibrosis transmembrane conductance regulator; C18: a CFTR corrector; p-hAQP1: plasmid encoding hAQP1; VX770, a potentiator of CFTR.

Substantial and non-conflicting data support the role of AQPs as one the mains actors involved in the sicca syndrome resulting from SS. It seems therefore logical that the modulation of AQPs mainly involved in SS is of paramount importance as a potential therapeutic target. Following this line of thought, several forays relating to AQP modulation in SS have been performed. In addition, several therapeutic approaches improving xerostomia have been shown to modulate AQP expression and/or trafficking. The role of AQPs in the treatment of xerostomia in SS are discussed hereafter and summarized in Figure 3.

### 5.1. Drug Therapies

Upon nerve stimulation, activation of M3 and M1 muscarinic receptors by acetylcholine leads to the activation of multiple intracellular pathways contributing to saliva secretion. Indeed, muscarinic receptor activation induces intracellular calcium increase, the activation of the Na^+^-K^+^-2Cl^−^ cotransporter, the activation of potassium channels, the activation of store-operated calcium channels (including Orai1 and transient receptor potential-canonical 1 (TRPC1)), the activation of the stromal activation molecules 1 and 2 (STIM1, STIM2) modulating Orai1 and TRPC1, the interaction between the transient receptor potential (TRP) channel vanilloid 4 (TRPV4) (activated by an inositol 1,4,5-triphosphate (IP3)-dependent mechanism and protein kinase-C dependent mechanism), the calcium-activated chloride channel anoctamin 1 (ANO1) (activated by calcium), and the activation of anion exchanger (Ae4; Slc4a9) upon simultaneous ß-adrenergic receptor stimulation [21,22,74,75,76,77]. Medications promoting the activation of one of the aforementioned intracellular pathway will improve xerostomia of various origins, including primary SS (pSS). As muscarinic receptor activation has also been shown to induce a calcium-dependent AQP5 intracellular trafficking to the apical membrane of acinar cells [78,79], medications aiming at promoting AQP5 protein expression and/or trafficking should also be beneficial for the treatment of xerostomia.

M3 and M1 muscarinic receptors agonists, as well as citric and malic acids, acidifying the oral environment and inducing saliva secretion through stimulation of taste buds and parasympathetic pathways, are expected to improve xerostomia. Several controlled randomized clinical trials that have analyzed the efficacy of cevimeline, an M1 and M3 receptor agonist, in SS patients or head and neck irradiated patients indicated that the drug could improve saliva flow in most cases [80,81]. Other clinical trials have shown the benefits of using pilocarpine, another M1 and M3 muscarinic receptor agonist, to treat xerostomia in SS patients and head and neck irradiated patients [80,82]. Three-time daily intake of 30 mg of cevimeline or of three- to four-time daily intake of pilocarpine represent therapeutic options, with limited side effects, for the treatment of xerostomia in SS patients and head and neck irradiated patients [80]. At a molecular level, cevimeline was shown to prevent radiation-induced xerostomia and radiation-induced decrease in *AQP5* expression in mice submandibular glands [83], and normalized AQP5 localization to the apical membrane of SG acinar cells in SS mice [84,85]. The effects of bethanechol, a carbamate ester resistant to cholinesterase, acting on M3 muscarinic receptors has been tested in patients with radiotherapy-induced xerostomia, but additional studies are required to assess the beneficial effects of the drug in the treatment of saliva hypofunction of other origins, including pSS [86]. As such, muscarinic agonists are likely improving xerostomia by activating multiple intracellular signaling pathways involved in saliva secretion (see above). Although randomized and controlled clinic trials investigating the effects of citric and malic acids in patients suffering from xerostomia showed improvement in saliva secretion, further studies using larger number of patients and longer follow-up times are required to further assess the beneficial effects of these compounds [80]. Furthermore, the effects of drug therapies on AQP5 protein expression and localization in irradiated and SS patients, and mice models, remain to be further documented.

More recently, the administration of VX770, a potentiator of cystic fibrosis transmembrane conductance regulator (CFTR), and C18, a CFTR corrector, were shown to partly restore AQP5 protein expression and transepithelial water efflux upon carbachol stimulation within SG from a NOD mice, a SS mouse model [86]. Furthermore, VX770 and C18 reduced salivary gland inflammation and restored saliva secretion in NOD mice and C18 restored saliva secretion in mice overexpressing BMP 6 (another SS mouse model) [86]. The usefulness of C18 and VX770 for the treatment of SS xerostomia requires deeper investigations. Finally, to our knowledge, no chemical modulator of AQP5 activity has yet been identified.

### 5.2. Gene Therapy

Due to their easy access requiring relatively noninvasive procedure and their encapsulation limiting vector spreading, SG present many advantages for gene delivery [87]. As such, *AQP1* gene therapy has been advocated to improve salivary secretion in irradiated-injured SG. Indeed, AQP1 gene transfer was hypothesized to facilitate water movement into ductal lumen across ductal cells, according to the speculated presence of an osmotic KHCO_3_ gradient (luminal > interstitial) [88]. Adenoviral-mediated *hAQP1* gene transfer to irradiated rat SG restored salivary secretion to a level close to that measured in sham-irradiated animals [88], likely by increasing water permeability in transduced acinar and subsequent enhanced muscarinic agonist-stimulated cell shrinkage [89]. Adenoviral-mediated *hAQP1* gene transfer [90] and adeno-associated viral (AAV2)-mediated *hAQP1* gene transfer [91] restored salivary secretion from irradiated minipig parotid glands. Although adenoviral-mediated *hAQP1* gene transfer to non-human primate SG was well-tolerated, its functional utility in enhancing fluid secretion from irradiated parotid glands was inconsistent [92]. Nevertheless, phase I clinical trial reported that in patients with salivary hypofunction resulting from radiation therapy, the administration of adenoviral vector encoding hAQP1 was able to induce rapid (within 42 days) saliva secretion improvement, in five patients out of the eleven patients included [93], that persisted even 3 to 4.7 years post-administration [94]. While modest systemic cell-mediated immune reactivity (2–3 fold) did not preclude responses to gene transfer within the five responding patients, a more important change impeded the efficacy of gene transfer in the remaining patients [95]. Despite its overall safety, the use of AdhAQP1 appears limited by the host immune response. A Phase I clinical trial evaluating the safety of AAV2hAQP1 delivery to parotid gland in patients suffering from irradiation-induced hypofunction is currently under way (NCT02446249). Ultrasound-assisted non-viral gene transfer of plasmid DNA coding for hAQP1 to irradiated minipig parotid gland restored salivary secretion [96]. Furthermore, as opposed to adenoviral vectors, low-intensity pulse ultrasounds delivery of plasmid DNA did not induce local inflammation [97]. Therefore, ultrasound-assisted non-viral gene transfer of hAQP1 to irradiated salivary glands might pave the way for new therapeutic options for patients suffering from xerostomia [96]. In a SS mouse model induced by *BMP6* over expression, *AQP1* gene therapy not only restored salivary and lacrimal gland secretion, but also decreased local and systemic inflammation, as shown by significant reductions in pro-inflammatory cytokines [98]. Despite the fact that *hAQP1* gene therapy represents an attractive option for the treatment of radiation-induced salivary gland hypofunction, further studies will be necessary to prove its usefulness for the treatment of xerostomia in SS patients. Other gene therapies aiming at transferring sonic hedgehog [99] or heat shock protein 25 [100] were shown to both restore *AQP5* expression and salivary secretion in irradiated mouse SG [101]. It still remains to be assessed whether other gene therapies correcting salivary hypofunction in SS mice models are capable of restoring *AQP5* expression/localization with SG. In any case, further studies will be required to determine if these additional gene therapies might be useful for the treatment of xerostomia in irradiated and SS patients. Overall, new viral vectors or alternative gene delivery methods, aiming at both maximizing the safety and efficacy of gene transfer, will be necessary to overcome the current limitations of gene therapy.

### 5.3. Ultrasound Therapies

Low-intensity pulse ultrasounds could represent an alternative way for the treatment of xerostomia. Indeed, in SS mice, low-intensity pulse ultrasounds improved both salivary secretion and AQP5 protein expression by reducing inflammation [97]. It remains to be determined whether low-intensity pulse ultrasounds could represent a useful therapy to treat xerostomia in irradiated and SS patients.

### 5.4. Regenerative Therapies

Cell differentiation from stem cells, present in intercalated ducts from SG, can explain the constant basal proliferative activity of the glandular epithelium [102]. Renewal of differentiated acinar cells relies on self-duplicating rather than on stem cell differentiation [102]. Several regenerative strategies have been proposed to restore salivary gland function, including the transplantation of autologous salivary gland-derived epithelial stem cells, the transplantation of non epithelial cell types (such as bone marrow-derived cells, bone marrow-derived mesenchymal stem cells, human adipose-derived mesenchymal stem cells, salivary-gland derived mesenchymal stem cells-like cells, amniotic cells, embryonic stem cells, induced-pluripotent stem cells, and dedifferentiated fat cells), the administration of bioactive compounds (such as growth factors, anti-apoptotic factors, angiogenic factors…), and the transplantation of salivary gland 3D tissue organoids [103,104,105,106,107]. Regenerative cell-based therapies aiming at increasing the number of differentiated acinar cells possessing proper AQP5 protein expression and localization necessary for saliva secretion have significantly progressed. They represent promising near future approaches for the treatment of radiotherapy-induced xerostomia and hyposalivation in patients suffering from SS.

## 6. Conclusions

The involvement of AQPs in the pathogenic process underlying SS is paramount. Altered distribution, localization, and maybe function (blocked in some cases by autoantibodies) of AQP5 in the salivary glands of SS patients, as well as the presence of auto antibodies in the sera of SS patients, support the role of AQP in SS. Indeed, in SS patients, autoimmune underpinnings account for combined mechanisms responsible for decreased saliva secretion. Salivary gland destruction and altered AQP5 localization, both induced by inflammation, are likely responsible for decreased water secretion. In addition, autoantibodies against AQPs and muscarinic receptors may alter the function of these proteins, and consequently, the mechanisms responsible for saliva secretion. Modulation of AQP and aquaporin gene transfer in patients could be a potential therapeutic option for restoring gland function and relieving the handicap resulting from sicca syndrome.

## Abbreviations

AQP	Aquaporin
pSS	Primary Sjögren’s syndrome
SG	Salivary gland
SS	Sjögren’s syndrome
